# Life course programming of stress responses in adolescents and young adults in India: Protocol of the Stress Responses in Adolescence and Vulnerability to Adult Non-communicable disease (SRAVANA) Study

**DOI:** 10.12688/wellcomeopenres.14583.1

**Published:** 2018-05-10

**Authors:** GV Krishnaveni, Kalyanaraman Kumaran, Murali Krishna, Sirazul Sahariah, Giriraj Chandak, Sarah Kehoe, Alexander Jones, Dattatray Bhat, Vijay Danivas, Krishnamachari Srinivasan, J Suguna Shanthi, SC Karat, Mary Barker, Clive Osmond, Chittaranjan Yajnik, Caroline Fall

**Affiliations:** 1 Epidemiology Research Unit, CSI Holdsworth Memorial Hospitol, Mysore, India; 2 Centre for the Study of Social Change, Mumbai, India; 3 Centre for Cellular and Molecular Biology, Hyderabad, India; 4 MRC Lifecourse Epidemiology Unit, University of Southampton, Southampton, UK; 5 Department of Paediatrics, University of Oxford, Oxford, UK; 6 Diabetes Unit, KEM Hospital Research Centre, Pune, India; 7 Mysore Medical College and Research Institute, Mysore, India; 8 St. John's Research Institute, St. John's National Academy of Health Sciences, Bangalore, India; 9 Department of Pediatrics, Jerudong Park Medical Centre, Jerudong Park, Brunei

**Keywords:** stress response, adolescents, TSST, India, non-communicable disease, maternal nutrition

## Abstract

**Background:** Early life nutrition may affect individuals’ susceptibility to adult non-communicable diseases (NCD). Psychological stress is a well-recognised NCD risk factor. Recent evidence suggests that impaired foetal nutrition alters neuro-endocrine pathways, and hypothalamic-pituitary-adrenal axis feedback systems, resulting in abnormal stress responses, and NCD risk. This study aims to examine adolescent cortisol and cardiovascular stress responses in relation to maternal nutrition and contemporaneous NCD risk markers.

**Methods**: The study sample will be drawn from three well-established birth cohorts in India; the Parthenon cohort, Mysore (N=550, age~20y), the SARAS KIDS prenatal intervention cohort, Mumbai (N=300, age~10-12y) and the Pune Rural Intervention in Young Adults/ PRIYA cohort, Pune (N=100, age~22y). We will perform the ‘Trier Social Stress Test (TSST)’, a well-accepted stress-test module which involves participants performing 5-minutes each of public speaking and mental arithmetic tasks in front of unfamiliar ‘judges’ (stressor). Repeated measures of salivary cortisol and autonomic cardiovascular outcomes relative to the stressor will be assessed. Measures of psychological stress, cognitive function, blood pressure, glucose-insulin metabolism and depression will be carried out. Mechanistic studies including DNA methylation in gluco-corticoid receptor ( *NR3C1*) and *11β-HSD2* gene loci and neuroimaging will be carried out in a subsample. Qualitative interviews and focus group discussions in a subsample of the Parthenon cohort will explore the perception of stress and stressors among the youth.

We will convert repeated measures into time-weighted averages before analysis. We will carry out multivariable regression analysis to test the associations. We will further refine the analyses using the mixed-model regression and conditional analyses for the association with repeated measures.

**Ethics and dissemination:** This study has been approved by the research ethics committee of CSI Holdsworth Memorial Hospital, Mysore. The findings will be disseminated locally and at international meetings, and reports will be submitted to open access peer reviewed journals.

## Introduction

Psychological stress is an unpleasant subjective experience, caused by an awareness of situational demands exceeding an individual’s adaptive capacity ^1^. It has been identified as an important lifestyle risk factor for adult non-communicable disease (NCD) ^2^. During stress, the body triggers physiological changes through the actions of the hypothalamic-pituitary-adrenal (HPA) axis (and its end product cortisol) and the autonomic nervous system, to maintain homeostasis ^2^. It is suggested that repeated psychological stress induces dysregulated HPA axis activity and autonomic cardiovascular control, reflected in either exaggerated or blunted responses of these systems to acute stress. Over time, these abnormalities can provoke the cardiometabolic, neuroendocrine and immunological disorders responsible for NCDs. Studies have shown increased cardiovascular and mental health disorders in relation to abnormal stress responses ^3, 4^.

The “Developmental Origins of Health and Disease (DOHaD) hypothesis” proposes that variations in nutrition during foetal development influence individual’s susceptibility to adult NCDs ^5^. Both intra-uterine growth restriction (resulting in low birthweight), and accelerated growth caused by maternal gestational diabetes (GDM), increase the risk of NCDs ^6– 8^. This phenomenon is thought to reflect permanent effects (‘programming’) of unbalanced foetal nutrition on physiological systems ^9^. Recent evidence suggests that altered neuro-endocrine responses to stress may be a pathway linking early life nutrition to the development of NCDs in later life ^10^. Maternal nutrients, particularly those involved in the one-carbon metabolism are vital for many neuro-developmental processes, and their deficiencies may alter neuroendocrine structure and function ^11, 12^, impair HPA axis feedback systems ^13, 14^, and influence stress reactivity. A few studies in adults and children have found associations between lower birth weight (as a proxy for maternal undernutrition) and higher cortisol and cardio-sympathetic responses to stress ^15– 18^. However, relationships of maternal nutritional status and maternal hyperglycemia *per se* with offspring stress responses are not known.

There is an escalating prevalence of NCDs in India; ~80 million people in the country are expected to develop type 2 diabetes by 2030 ^19^, and the estimated prevalence of GDM is ~15% currently ^20^. With recent socio-economic transition, resulting in energy dense diets and physical inactivity, these conditions are increasingly affecting younger age groups ^21^. Recent reports suggest that stress levels are also increasing steadily among youth, resulting in a growing prevalence of anxiety disorders, depression and suicide ^22, 23^. In India, widespread maternal undernutrition, with specific micronutrient deficiencies, as well as high rates of maternal GDM, may confer long-term cardiometabolic and neuro-cognitive adverse effects on the offspring ^24^. However, there is limited evidence for the early life programming of stress responses as a risk factor for NCDs in India. Our own studies at Holdsworth Memorial Hospital (HMH), Mysore showed that increased HPA axis activity, measured using fasting plasma cortisol concentration, was associated with higher cardiometabolic risk markers in adults and children ^25, 26^. Subsequently we observed that the adolescent offspring of GDM mothers (OGDM) exhibited exaggerated cardiovascular responses to acute stress ^27^.

Adolescence, the developmental period from the onset of puberty until the attainment of social independence, is associated with intense negative emotions, stress perception and reactivity ^28^. These attributes are thought to result from extensive re-modelling of cortical brain structure and changing efficiency of cognitive and emotional control occurring during this period ^28^. Stress-related behavioural and physiological changes during adolescence and early adulthood may initiate risk trajectories for NCDs, and may also have adverse implications for future offspring through programming effects, thus perpetuating the risk cycle. Various biological, environmental and social factors may determine variations in stress-reactivity ^29^. Understanding these factors, critical life course periods during which they influence changes in stress responses, and mechanisms underlying these changes may lead to interventions to mitigate their adverse effects on health. The current study proposes to build on the previous evidence to understand the life course factors that predict adolescent stress responses.

## Aims and objectives

The overarching aim of the study is to understand modifiable factors that might reduce stress responses and guide the development of an integrated intervention to reduce stress reactivity, and therefore future risk of NCDs in adolescents and young adults in India.

The key objectives are to:

1. Test the persistence of the association of birth weight, and maternal GDM and micronutrient status with cortisol and autonomic cardiovascular responses to acute stress in adolescents
2. Examine the association of stress responses with their psychological and cardiovascular risk markers
3. Test the role of prenatal nutritional intervention in reducing physiological stress responses in the adolescent offspring
4. Test whether nutritional supplementation during adolescence/young adulthood is associated with improved stress responses in young adults exposed to maternal nutritional deficiency
5. Investigate specific epigenetic (methylation) changes, and neurocognitive characteristics that relate to stress responses, and
6. Develop a socio-cognitive intervention model to be used in a future study to reduce stress responses in adolescents and young adults

## Protocol

### Study sample

The “Stress Responses in Adolescence and Vulnerability to Adult Non-communicable disease (SRAVANA) study” will adopt a multi-centric approach, leveraging on the unique characteristics of three Indian birth cohorts to achieve specific, but complementary objectives.

**The Mysore Parthenon Cohort** was established at HMH in 1997–1998 to investigate the long-term effects of maternal GDM and nutritional status on offspring NCD risk ^30^. The cohort comprised 663 normal offspring of 830 women whose GDM status had been assessed; ≈6% of them were OGDM ^31^. Offspring follow-up continued every 6–12 months after birth for anthropometry, body composition, pubertal staging, cardiometabolic and cognitive assessments. The study showed high rates of low maternal B12 and vitamin D status, and GDM, suggesting that undernutrition and overnutrition co-exist in this transitioning population ^31– 33^. Offspring exposed to maternal GDM had greater adiposity, insulin resistance and systolic BP, and higher cardiovascular stress responses during adolescence than those of mothers with normal glucose tolerance ^27, 34^. Higher risk markers were also observed in relation to lower offspring birthweight and maternal nutritional imbalances (vitamin D, folate and homocysteine) ^32, 33, 35^.

The cohort participants are currently aged 20, and ≈85% of the original cohort are still being followed up (N=550). For the present study, we will measure NCD risk markers, chronic stress in the whole cohort, and acute stress responses in a subsample (N~250) who participated in earlier studies of stress reactivity at the age of 13.5 years ^27^. See below for a detailed description of these assessments.

**The Mumbai Maternal Nutrition Project** (MMNP or “Project SARAS”; ISRCTN 62811278) was a community-based randomised trial of nutritional supplementation for women living in urban slums, pre-conceptionally and throughout pregnancy ^36^. The primary outcome was birth weight, but the study was designed to have enough power to study long-term effects on body composition, cardiometabolic risk markers and cognitive function in the children. The intervention was a daily snack (such as samosas and fritters) made from micronutrient-rich foods (milk, green leafy vegetables and fruit). Women in the control group received a similar snack made from foods of lower micronutrient content such as potato and onion. A total of 6513 women were randomised, 2291 women became pregnant, and 1962 delivered live singleton newborns during 2006–2012. The study showed that supplementation increased offspring birthweight among women of normal or high body mass index ^36^. The incidence of GDM was halved in the intervention mothers compared to controls ^37^.

SARAS KIDS is the ongoing follow-up study of the offspring born to these women. In the SRAVANA study, we will randomly select 150 offspring each from the maternal intervention and control groups and all available OGDM during early adolescence (~10–12y). We will measure NCD risk markers and stress responses. We will test the hypothesis that ‘prenatal nutritional supplementation normalises cortisol and cardiovascular stress responses, especially in OGDM’.

**The Pune Maternal Nutrition Study (PMNS)** was a community based observational study set up in rural Pune in 1993 ^38^. Non-pregnant, married women were identified, and followed up until they became pregnant (N=797). Pregnant women were assessed for diet, micronutrient status and foetal growth. The 753 offspring formed the PMNS cohort, and had detailed anthropometry at birth and at 6-monthly intervals. Maternal undernutrition (low BMI and vitamin B12 status) was widespread in this population. Low B12 status in the mothers was associated with higher insulin resistance in the children.

The PMNS offspring are now part of a community-based intervention study (ISRCTN 32921044), with the hypothesis that physiological doses of micronutrient supplementation during adolescence/young adulthood will improve the birthweight, B12 status, and newborn and childhood body composition in the next generation ^39^. The cohort members (N=557) were enrolled when they were ~16–18y old, and were randomised to receive either i) B12 2µg, ii) B12 2µg plus multiple micronutrients plus milk protein (MMN), or iii) placebo daily. The intervention is ongoing. We will recruit ~50 participants each from the intervention and placebo groups at 22–23y of age. We will measure NCD risk markers and stress responses. We will test the hypothesis that current micronutrient supplementation is associated with improved cortisol and cardiovascular responses to stress in young adults exposed to maternal nutritional deficiencies.

### Assessments ( Table 1)

**The Trier Social Stress Test (TSST)** is a valid method of ethically inducing acute psychological stress, particularly in adolescents ^40^. The test is based on ‘uncontrollability, unpredictability and social evaluative threat (perception of negative assessment of the self by others)’ for inducing stress. We have previously adapted the TSST modified for children (TSST-C) for use in the Parthenon adolescents, and have shown that it is valid for Indian conditions ^41^. The TSST involves participants performing 5-minutes each of public speaking and mental arithmetic tasks in front of unfamiliar evaluators (stressor). Pre-test and post-test saliva samples will be collected for cortisol assessment. Systolic and diastolic blood pressure (BP), cardiac output, stroke volume, heart rate and total peripheral resistance, as measures of autonomic nervous system, will be measured continuously before and during the TSST using a portable hemodynamic monitor (Nexfin, BMEye). Acute stress response will be estimated as the change in post-stress cortisol and cardiovascular parameters from pre-test values (baseline). These measures form the primary outcome of the study.

**Table 1.** List of assessments and outcomes of the proposed study.

Parameters tested	Method	Outcome	Mysore	Mumbai	Pune
**Stress response**	Trier Social Stress Test	Cortisol concentration; continuous blood pressure, heart rate, cardiac output, stroke volume and total peripheral resistance Cortisol and cardiovascular stress response	✓	✓	✓
**Cardiometabolic** **Parameters**	Anthropometry	Weight, height; mid-upper arm, head, waist and hip circumference; subscapular and triceps skinfold	✓	✓	✓
	Body composition (bioimpedance)	Body fat and lean mass	✓	✓	✓
	Hand grip strength (dynamometer)	Muscle strength	✓	✓	✓
	Blood pressure	Resting systolic and diastolic blood pressure; pulse rate	✓	✓	✓
	Oral glucose tolerance test	Diabetes/glucose intolerance	✓	☑	☑
	Fasting blood samples	Fasting insulin for insulin resistance (HOMA-IR); total cholesterol, triglycerides, HDL-cholesterol	✓	☑	☑
**Psychological** **Parameters**	Wechsler’s Adult Intelligence Scale-IV	Cognitive function	✓	☑ *	☑
	PHQ-9	Depression	✓	-	✓
	MINI		✓	-	☑
**Behavioural** **Parameters**	Perceived Stress Scale	Chronic stress	✓	-	✓
	Stressful Life Events Scale		✓	-	✓
	Strengths and Difficulties Questionnaire	Internalising and externalising behaviour	✓	✓	✓
**Lifestyle** **Indicators**	Food Frequency Questionnaire	Dietary intake pattern Nutrient intake quantity	✓	✓	☑
	24-hour Food Recall		✓	✓	✓
	International Physical Activity Questionnaire	Physical activity pattern, intensity and magnitude	✓	✓	✓
	Smoking and Alcohol intake	Addictive behaviour, substance Abuse	✓	-	✓
**Mechanistic** **Assays**	Epigenetic Assay	DNA methylation: *NR3C1, 11β-HSD2*	✓	✓	-
	Magnetic Resonance Imaging for brain	Regional cerebral volumes	✓	✓	☑
**Socio-** **demographic** **Indicators**	Standard of Living Index	Socio-economic Status	✓	✓	✓

✓: SRAVANA study assessments; ☑: Assessed as part of ongoing/recently completed follow-up. HOMA-IR: Homeostasis Model Assessment for Insulin Resistance; MINI: Mini International Neuropsychiatric Interview; PHQ-9: Patient Health Questionnaire-9 *Kauffman’s Assessment Battery and additional tests

### Secondary outcomes

**Psychological assessments:** We will administer standard questionnaires, valid for Indian adults/adolescents for psychological measures of chronic stress and behavioural assessment.

1) Perceived stress scale ^1^, a commonly used 14-item scale to measure the degree to which situations in one’s life during the past month are appraised as stressful, and gives a measure of chronic stress.

2) Stressful Life Events Scale is a 40-item scale to assess chronic stress caused by life events among participants in the past one year ^42^.

3) Strengths and Difficulties Questionnaire, a behavioural screening questionnaire for children and adolescents.

4) Depression and other psychological health issues will be identified using the Patient Health Questionnaire-9 (PHQ-9) and MINI (Mini International Neuropsychiatric Interview) ^43, 44^. The PHQ-9 is a screening questionnaire for depression, and gives a score that measures severity of depression, while MINI is a diagnostic interview schedule for diagnosing depression.

5) Cognitive function will be assessed in Mysore, and Pune (as part of ongoing follow-up), using Wechsler Adult Intelligence Scale-Fourth Edition - India (WAIS-IV ^India^) a widely used standard IQ test designed to measure intelligence and cognitive ability in adults and older adolescents ^45^. In Mumbai, cognitive function has been recently assessed for the SARAS KIDS children using three core tests from the Kauffman’s Assessment Battery and some additional tests ^46– 48^.

**Cardiometabolic assessments:** We will measure weight (digital scale), height (adult stadiometer), head, mid-upper arm, waist and hip circumferences (anthropometric tape), subscapular and triceps skinfold thickness (Harpenden callipers), hand-grip strength (Jamar dynamometer), and body composition (bioimpedance). Waist-to-hip ratio will be calculated for central adiposity. Resting blood pressure will be measured on the non-dominant arm using an automatic device after the participants have been seated relaxed for five minutes.

An oral glucose tolerance test ( WHO protocol) will be administered in Mysore after an overnight fasting for plasma glucose, insulin and lipid concentrations. In Mumbai and Pune OGTTs using the WHO protocol have been carried out recently as part of ongoing follow-up.

**Lifestyle indicators:** Dietary intake will be assessed using a purpose-designed food frequency questionnaire (FFQ) ^49^ and a 24-hour recall questionnaire ( Supplementary File 1 and Supplementary File 2). Physical activity will be measured using the International Physical Activity Questionnaire – Short version. Information on smoking and alcohol intake will be documented. 

**Laboratory assays:** All assays will be carried out using standard protocols. Salivary samples for cortisol assay will be collected using synthetic swabs (Salivette ^®^Cortisol, Sarsteadt, Germany). Samples will be stored at -20° till analysis.

A part of the whole blood sample at all time points (EDTA vacutainer) will be transferred to 2 ml micro centrifuge tubes for a hemogram. The remaining blood will be centrifuged (2500 g × 15 min) within 30-minutes of collection, and the separated plasma stored at -80°C until further analysis. Packed cells will be stored for DNA.

Biochemistry analyses will be undertaken for various measurements:

• Hemogram: Cell Counter hematology analyser on the day of collection.
• Plasma glucose and lipids: Automated biochemistry analyser (Hitachi 902, Roche Diagnostics, Germany) using standard enzymatic kits.
• Insulin: ELISA kit (Mercodia AB, Uppsala, Sweden).
• Cortisol: ELISA kit (Alpco Diagnostics, Salem, NH)

**Epigenetic assays:** In the Parthenon and SARAS KIDS cohorts, we will use a candidate gene approach to assess epigenetic characteristics (DNA methylation) at the gluco-corticoid receptor ( *NR3C1*) and *11β-HSD2* gene loci in relation to maternal factors and offspring stress responses, and explore the effect of maternal nutritional supplementation on these associations. Studies in humans and animals have shown that methylation levels in the promoter regions of these genes may be influenced by maternal GDM and/or undernutrition and have adverse implications for offspring stress responses ^50– 52^.

**Neuroimaging:** We will collect the following Magnetic Resonance Imaging (MRI) biomarkers in subsamples of participants in Mysore and Mumbai (N=100 each): 1) whole brain, hippocampal and amygdale volumes and cortical thickness and surface area, 2) cortical structural complexity measured using fractal dimension from volumetric images, 3) white matter integrity quantified by fractional anisotropy, radial diffusivity and mean diffusivity from diffusion tensor imaging and 4) functional connectivity of networks quantified from resting state fMRI.

T1-weighted volumetric sequences will be acquired for the assessment of brain regional volumes, cortical thickness, cortical surface area, gyrification index, fractal dimension, voxel base morphometry analysis.

Neuroimaging data will be investigated with the aim of identifying significant correlations of cortisol and cardiovascular stress responses with structural and functional brain networks.

**Other:** Information will be collected on medical and drug history, recent stressful events, education, occupation and socio-economic status, and parental diabetes and cardiovascular disease.

### Intervention development

We propose to develop a socio-cognitive intervention method for stress management. We intend to use a person-based approach to develop a culturally-appropriate intervention design within the overarching framework of the MRC guidance on complex intervention development ^53, 54^. ‘A person-based approach to intervention development’ aims to understand, and accommodate, the perspectives and psychosocial context of the intervention users in a systematic manner, through qualitative research, thus strengthening evidence-based interventions.

We will carry out three distinct pieces of work that will run parallel with the other components as below.

**i) Systematic review:** will be carried out to understand what works in reducing perceived stress in adolescents and young adults. We will follow the Preferred Reporting Items for Systematic Reviews and Meta-Analyses ( PRISMA) statement ^55^.

Eligibility criteria, search strategy and identification of literature: We will consider any non-therapeutic psychological interventions as our exposure of interest. Outcome will be ‘perceived stress’ in adolescents and young adults. We will include all intervention studies published in English until the start of the review process. We will search Medline/PubMed and the Cochrane Library databases using the medical subject headings (MeSH) terms and text word terms and reference lists of retrieved literature.

Data extraction and quality assessment: Data extraction and quality assessment of each article will be carried out independently by GVK and another assessor. Discrepancies between assessors will be resolved by discussion with a third independent assessor.

Evidence synthesised from this will inform the content and delivery form of the intervention.

**ii) Formative qualitative research**: We will conduct qualitative interviews with ~25 adolescents and young adults in Mysore, soliciting their views on stresses and stressors that affect their daily life, and the ways to tackle them. Specific suggestions from the participants for activities and techniques to be used in the intervention design will be discussed (eg. digital sources of support). Analysis of the pooled data from this work will identify ‘guiding principles’ on the key issues that must be addressed by the intervention, and specific ideas about the most effective types of support that could be offered.

**iii) Development of intervention format and content:** This work will produce 1) a co-created set of intervention materials that are ready to be tested, and 2) a decision on the intervention delivery format. To understand intervention users’ perspective of what might or might not be helpful in reducing stress, we will conduct ‘focus group discussions’ with 8–10 adolescents/ young adults to explore intervention formats (eg. smartphone-based) and content (eg. applied techniques) based on existing resources as means of support for managing stressful situations. Participants will also be invited to discuss their experiences of using any other resources.

A set of intervention materials will be produced based on the results of the above three steps. **‘Think aloud’** interviews will be conducted where participants will be asked to verbalise their thoughts as they go through the intervention materials, which will identify features and technologies that they particularly enjoy and think they will use, and those that would irritate them ^56^. Materials will be adapted iteratively between interviews. In a longitudinal study, 5–10 young adults will be asked to use the intervention materials for several weeks before being interviewed about their experiences.

The final intervention design will incorporate the full range of techniques known to be effective, and identified by young adults as being acceptable, in reducing and helping to manage stress. The intervention module will be pilot tested in a future study.

### Data analysis

We will summarise repeated measures of cortisol and cardiovascular response to stress in a time weighted average (TWA). Linear and logistic regression models will be used to examine associations of stress responses (TWA) with risk outcomes (eg. blood pressure, depression). These models will be adjusted for age, sex, socio-economic status, and relevant maternal and offspring co-variates.

We will use linear mixed-model regression analysis to examine associations of maternal exposures with repeated measures of cortisol and cardiovascular parameters to account for within-group correlations.

We will use the conditional analysis method ^57^ to examine the change in stress responses from early adolescence to adulthood in Mysore, to explore tracking.

Metanalysis: We will also do metanalyses of data from Mysore, Mumbai and Pune components of the study on the association of stress responses with 1) maternal nutritional status and 2) offspring NCD risk markers.

Qualitative interviews: Transcripts from qualitative interviews and conversations will be subject to thematic analysis, using a standard thematic analysis methodology involving constant comparative methods.

### Sample size and power calculation

Mysore: The sample size of the Mysore study component is based on the previous stress-responses study carried out during 2011–2013 among the Parthenon cohort participants ^41^. The previous sample size was calculated based on the only available study in children on the association between birth weight and stress response ^15^. We estimated that our sample will have 80% power to detect an effect size of 2.9 nmol cortisol increase per kg increase in birth weight at 5% significance level. In the current study, we aim to follow-up all the children who had participated in the above study. This sample size will have 80% power at the 5% significance level to detect an association of 0.18 SD of a continuous outcome (eg. TWA salivary cortisol) per SD of an exposure (eg. birth weight).

Mumbai: The sample size calculation for the Mumbai component of the study is based on the Mysore sample size. The estimated sample size will be able to detect an association of 0.16 SD of a continuous outcome per SD of a continuous exposure, and will detect a difference in outcome of 0.23 SD between the intervention and the control arms.

Pune: As there are no earlier studies on the effect of current nutritional intervention on stress responses, we based the sample size for this study on the findings from comparison of effect size between the offspring of B12 deficient and normal B12 status mother. The estimated sample size will detect an association of 0.28 SD of a continuous outcome per SD of an exposure. The study will detect a difference of 0.56 SD in the outcome between the control and intervention group.

### Ethics and dissemination

We follow Indian Council of Medical research (ICMR) guidelines for the ethical conduct of biomedical research. We have already received approval for the entire study from the ethics review committee of CSI Holdsworth Memorial Hospital, the host institution. Ethical permission will be sought additionally from the institutional ethical review committees in Mumbai and Pune prior to the start of these study components.

The participants will be given written information and the study is explained verbally, either telephonically or by home visit by research assistants/health workers. The participants will be encouraged to ask questions before obtaining consent. Written informed consent will be obtained for adult participants, and from parents in case of children <18 years (Mumbai) before commencing data collection. Verbal assent will be obtained from the children. Consent for genomic samples will be taken specifically.

The data generated from this study will be shared with the scientific community at local, national and international conferences, and published in open access peer review journals. The major findings will be shared with the study participants and their families in lay terms through public engagement initiatives.

### Study status

The follow-up of the Mysore Parthenon cohort participants is currently underway. By the end of April 2018, 82 participants have completed the TSSTs and 309 participants have undergone OGTT and assessments for other secondary outcomes except cognitive function. Qualitative interviews have been carried out among 5 adolescents/young adults.

## Conclusion

Altered neuro-endocrine reactivity to stressful situations is a risk factor for cardiovascular and mental health disorders in humans. Previous studies in India and other parts of the world have shown consistent associations between impaired early life nutrition and increased risk of these disorders. There is limited evidence for a role of early nutritional programming of stress responses, though this is considered one of the pathways for increased adult disease risk. Micronutrient deficiencies, especially of one-carbon nutrients, are associated with psychological manifestations of stress in humans ^58^. However, there are no studies examining the effect of nutritional supplementation on physiological stress responses in young individuals. Our study attempts to fill gaps in this area of research and explore in detail behavioural, environmental, epigenetic and neurological aspects linking early nutritional exposure to variations in stress reactivity.

To our knowledge, this is the first study to explore the role of life course nutrition on adolescent stress responses in humans. The study will generate robust evidence for the role of intra-uterine nutritional imbalances in the development of NCDs through programming of abnormal stress responses. Capitalising on the existing intervention designs in two well-known Indian birth cohorts, it will determine whether 1) prenatal nutritional supplementation and 2) current nutritional supplementation mitigates abnormal stress responses in vulnerable adolescents. The secondary analysis of these data, along with the existing past data, will guide the development of multi-faceted interventions for reducing NCD risks through a life course approach.

## Declarations

### Ethics approval

The study has been approved by the Ethics Committee of the CSI Holdsworth Memorial Hospital, Mysore (June 2017; no. CSIHMH/ERU2017/1). The committee membership follows Indian Council of Medical Research guidelines and is composed of a total of nine members (2 clinicians, 3 scientists, 1 legal expert, 1 social scientist/spiritual leader, 1 academician and 1 lay person).

## Data availability

All data underlying the results are available as part of the article and no additional source data are required

## Supplementary material

Supplementary File 1: Food frequency questionnaire.

Supplementary File 2: Food recall questionnaire.
