# Supplementary material for: Life course programming of stress responses in adolescents and young adults in India: Protocol of the Stress Responses in Adolescence and Vulnerability to Adult Non-communicable disease (SRAVANA) Study
Source: Wellcome Open Res. 2018 May 10;3:56. [Version 1] doi: 10.12688/wellcomeopenres.14583.1 (PMC6039920; doi:10.12688/wellcomeopenres.14583.1)
Supplement: Supplementary file 1 [file wellcomeopenres-3-15878-s0000.tgz › 44603daa-071d-4a4f-a814-d2534f368549.docx]

**FOOD FREQUENCY QUESTIONNAIRE**

##### TIME PERIOD

**The reference time period for this questionnaire is a TYPICAL MONTH.**

##### FREQUENCY

**D1 = Once daily, D2 = Twice daily, D3 = 3 Times daily etc.**

**W1 = Once weekly, W2 = Twice weekly, W3 = 3 Times weekly, W4 = 4 Times weekly etc**

**M1 = Once monthly, M2 = Twice monthly, M3 = 3 Times monthly**

**UNIT OF MEASURE - (See photographs)**

**B1 = Large bowl, B2 = Small bowl**

**G2 = Large glass, G3 = Small glass**

**P1 = Palya spoon**

**PL1 = Plate**

**S4 = Sambhar spoon, S5 = Saagu spoon,**

**TS1 = Large teaspoon**

**TS2 = Small teaspoon**

**NUMBER OF UNITS**

**Please enter whole numbers or decimals in this box (e.g. 0.5, 0.25 ). NOT fractions (e.g. 1/2,1/4 ).**

##### SIZE

**C = CIRCUMFERENCE 1-7**

Please don’t write in the grey boxes

| **SECTION A: BEVERAGES** | | | | | | |
| --- | --- | --- | --- | --- | --- | --- |
| **1** | **FRESH MILK, PACKET MILK SKIMMED, *SPECIFY BRAND*:** |  | **G3** |  |  |  |
| **2** | **TEA** |  | **G3** |  |  |  |
| **3** | **COFFEE** |  | **G3** |  |  |  |
| **4** | **HORLICKS, COMPLAN, BOOST, BOURNVITA, BADAM MILK** |  | G3 |  |  |  |
| **5** | **BUTTERMILK** |  | **G2** |  |  |  |
| **6** | **MIRINDA / PEPSI / SPRITE , LEMONADE (LEMON JUICE WITH SUGAR)** |  | **G2** |  |  |  |
| **7** | **FROOTY / MAZA** |  | **G2** |  |  |  |
| **8** | **KISAN/ RASNA/ ROOHAFZA** |  | **G2** |  |  |  |
| **9** | **FRESH FRUIT JUICE** |  | **G2** |  |  |  |
| **10** | **OTHER BEVERAGE 1, *SPECIFY*:** |  | **G2** |  |  |  |
| SECTION B: FRUIT | | | | | | |
| **1** | **APPLE** |  | **QUANTITY** |  | **S M L** |  |
| **2** | **BANANA (PACCHE)** |  | **QUANTITY** |  |  |  |
| **3** | **BANANA (YELAKKI)** |  | **QUANTITY** |  |  |  |
| **4** | **SWEET LIME** |  | **SEGMENT** |  |  |  |
| **5** | **GUAVA** |  | **QUANTITY** |  | **S M L** |  |
| **6** | **ORANGE** |  | **SEGMENT** |  |  |  |
| **7** | **POMEGRANATE** |  | **QUANTITY** |  | **S M L** |  |
| **9** | **SAPOTA** |  | **QUANTITY** |  | **S M L** |  |
| **10** | **GRAPES** |  | **QUANTITY** |  |  |  |
| **11** | **MANGO (SEASONAL)** |  | **QUANTITY** |  |  |  |
| **12** | **WATERMELON** |  | **SLICE** |  | **S M L** |  |
| **13** | **OTHER 1, *SPECIFY*:** |  |  |  |  |  |
| SECTION C: DRIED FRUIT / NUTS | | | | | | |
| **1** | **DATES** |  | **QUANTITY** |  |  |  |
| **2** | **RAISINS** |  | **QUANTITY** |  |  |  |
| **3** | **CASHEW NUT** |  | **QUANTITY** |  |  |  |
| **4** | **ALMOND** |  | **QUANTITY** |  |  |  |
| **5** | **CHOPPED MIXED DRYED FRUITS** |  | **QUANTITY** |  |  |  |
| **6** | **OTHER 1, *SPECIFY*:** |  |  |  |  |  |
| SECTION D: RICE FOODS | | | | | | |
| **1** | **PLAIN/BOILED RICE** |  | **B1** |  |  |  |
| **2** | **RICE WITH DHAL** |  | B1 |  |  |  |
| **3** | **TAMARIND RICE** |  | **B1** |  |  |  |
| **4** | **LEMON RICE** |  | **B1** |  |  |  |
| **5** | **RICE WITH ONION OR RICE WITH ONION + TOMATO** |  | **B1** |  |  |  |
| **6** | **GHEE RICE** |  | **B1** |  |  |  |
| **7** | **VEGETABLE BATH** |  | **B1** |  |  |  |
| **8** | **VEGETABLE BATH WITH GLV** |  | **B1** |  |  |  |
| **9** | **CURD RICE** |  | **B1** |  |  |  |
| **10** | **PLAIN IDLY** |  | **QUANTITY** |  |  |  |
| **11** | **PLAIN DOSA, DOSA MASALA, ONION DOSA** |  | **QUANTITY** |  | **C** | **T1 T2** |
| **12** | **PUFFED RICE** |  | **B1** |  |  |  |
| **13** | **RICE ROTI** |  | **QUANTITY** |  | **C** | **T1 T2** |
| **14** | **AVALAKKI / RICE FLAKES** |  | **B1** |  |  |  |
| **15** | **OTHER 1, *SPECIFY*:** |  |  |  |  |  |
| SECTION E: WHEAT FOODS | | | | | | |
| **1** | **CHAPATI** |  | **QUANTITY** |  | **C** | **T1 T2** |
| **2** | **POORI** |  | **QUANTITY** |  |  |  |
| **3** | **PARATA** |  | **QUANTITY** |  | **C** | **T1 T2** |
| **4** | **BUN / KARA BUN** |  | **QUANTITY** |  |  |  |
| **5** | **SLICED BREAD** |  | **SLICE** |  |  |  |
| **6** | **UPPUMA** |  | **B1** |  |  |  |
| **7** | **RAVA IDLY** |  | **QUANTITY** |  |  |  |
| **8** | **WHEAT VERMICELLI** |  | **B1** |  |  |  |
| **9** | **OTHER 1, *SPECIFY*:** |  |  |  |  |  |
| SECTION F: RAGI FOODS | | | | | | |
| **1** | **RAGI ROTI** |  | **QUANTITY** |  | **C** | **T1 T2** |
| **2** | **RAGI BALL** |  | **QUANTITY** |  |  |  |
| **3** | **RAGI DOSA** |  | **QUANTITY** |  | **C** | **T1 T2** |
| **4** | **OTHER 1, *SPECIFY*:** |  |  |  |  |  |
| SECTION G: VEGETARIAN PREPARATIONS | | | | | | |
| **1** | **POTATO PALYA** |  | **P1** |  |  |  |
| **2** | **VEGETABLE PALYA** |  | **P1** |  |  |  |
| **3** | **VEGETABLE SAAGU** |  | **P1** |  |  |  |
| **4** | **GLV / SOPPU PALYA** |  | P1 |  |  |  |
| **5** | **WHOLE GRAMS PALYA** |  | **P1** |  |  |  |
| **6** | **WHOLE SPROUTS PALYA** |  | **S4** |  |  |  |
| **7** | **TURDAL CURRY WITH OR WITHOUT VEGETABLES** |  | **S4** |  |  |  |
| **8** | **TURDAL CURRY WITH GLV** |  | **S4** |  |  |  |
| **9** | **WHOLE SPROUTS CURRY** |  | **S4** |  |  |  |
| **10** | **WHOLE GRAMS CURRY** |  | S4 |  |  |  |
| **11** | **WHOLE GRAM CURRY WITH GLV** |  | **S4** |  |  |  |
| **12** | **RASAM** |  | **S4** |  |  |  |
| **13** | **OTHER 1, *SPECIFY*** |  |  |  |  |  |
| SECTION H: RAW VEGETABLES | | | | | | |
| **1** | **CUCUMBER** |  | **SLICES** |  |  |  |
| **2** | **TOMATO** |  | **QUANTITY** |  |  |  |
| **3** | **ONION** |  | **QUANTITY** |  |  |  |
| **4** | **CARROT** |  | **QUANTITY** |  |  |  |
| **5** | **RADISH** |  | **QUANTITY** |  |  |  |
| **6** | **KOSAMBARI** |  | **B2** |  |  |  |
| **7** | **BEANS** |  | **QUANTITY** |  |  |  |
| **8** | **OTHER 1,*SPECIFY*:** |  |  |  |  |  |
| **SECTION I: PREPARATION (NON VEGETARIAN)** | | | | | | |
| **1** | **CHICKEN BIRIYANI** |  | B1 |  |  |  |
| **2** | **MUTTON BIRIYANI** |  | B1 |  |  |  |
| **3** | **EGG BIRIYANI** |  | **B1** |  |  |  |
| **4** | **KEEMA BALL** |  | **QUANTITY** |  |  |  |
| **5** | **KEEMA CURRY** |  | **QUANTITY** |  |  |  |
| **6** | **FISH** |  | **QUANTITY** |  |  |  |
| **7** | **CHICKEN (INCLUDING BIRIYANI)** |  | **QUANTITY** |  |  |  |
| **8** | **MUTTON (INCLUDING BIRIYANI)** |  | **QUANTITY** |  |  |  |
| **9** | **BOILED EGG** |  | **QUANTITY** |  |  |  |
| **10** | **EGG FRY** |  | **P1** |  |  |  |
| **11** | **OMELETTE** |  | **QUANTITY** |  |  |  |
| **12** | **OTHER 1, *SPECIFY*:** |  |  |  |  |  |
| SECTION J: JAM / CHUTNEY | | | | | | |
| **1** | **JAMS & JELLIES** |  | **TS2** |  |  |  |
| **2** | **HONEY** |  | **TS2** |  |  |  |
| **3** | **OTHER 1 *SPECIFY*:** |  | **TS2** |  |  |  |
| SECTION K: ADDED SUGAR | | | | | | |
| **1** | **SUGAR IN FRUIT JUICE** |  | **TS2** |  |  |  |
| **2** | **SUGAR IN TEA / COFFEE / MILK** |  | **TS2** |  |  |  |
| **3** | **SUGAR WITH DOSA** |  | **TS2** |  |  |  |
| **4** | **SUGAR WITH CHAPATI/ PURI** |  | **TS2** |  |  |  |
| **5** | **SUGAR WITH IDLY** |  | **TS2** |  |  |  |
| **6** | **SUGAR WITH ANY OTHER FOOD**  ***SPECIFY*:** |  | **TS2** |  |  |  |
| SECTION L: SAVOURY (SALTED) SNACKS | | | | | | |
| **1** | **PAPAD/ SANDIGE** |  | **QUANTITY** |  | **C** |  |
| **2** | **CHAKLI** |  | **QUANTITY** |  |  |  |
| **3** | **VEGETABLE SAMOSA** |  | **QUANTITY** |  |  |  |
| **4** | **PAKODA** |  | **QUANTITY** |  |  |  |
| **5** | **BENGAL GRAM WADA** |  | **QUANTITY** |  |  |  |
| **6** | **BLACK GRAM WADA** |  | **QUANTITY** |  |  |  |
| **7** | **KARA** |  | **B2** |  |  |  |
| **8** | **CHIPS (POTATO)** |  | **B1** |  |  |  |
| **9** | **CHIPS (PLANTAIN)** |  | **B1** |  |  |  |
| **10** | **PUFF** |  | **QUANTITY** |  |  |  |
| **11** | **SALTED BISCUIT** |  | **QUANTITY** |  |  |  |
| **12** | **OTHER 1, *SPECIFY*:** |  |  |  |  |  |
| SECTION M: SWEET SNACKS | | | | | | |
| **1** | **CHOCOLATE BAR**  ***SPECIFY BRAND*:** |  | **QUANTITY** |  |  |  |
| **2** | **CAKES & PASTRY** |  | **QUANTITY** |  |  |  |
| **4** | **PLAIN BISCUIT (SWEET)** |  | **QUANTITY** |  |  |  |
| **6** | **TOFFEE/ CANDY** |  | **QUANTITY** |  |  |  |
| **7** | **SWEETS** |  |  |  |  |  |
| **8** | **ICE CREAM** |  |  |  |  |  |
| **9** | **ICE LOLLY** |  |  |  |  |  |
| **10** | **OTHER SWEETS OR SNACKS, *SPECIFY*:** |  |  |  |  |  |
| SECTION N: FAST FOOD | | | | | | |
| **1** | **PANI PURI / MASALA PURI** |  | **PL 1** |  |  |  |
| **2** | **CHURMURI** |  | **B1** |  |  |  |
| **3** | **GOBI MANCHURIAN** |  | **PL 1** |  |  |  |
| **4** | **MAGI NOODLES** |  | **B1** |  |  |  |
| **5** | **PASTA & MACARONI** |  | **B1** |  |  |  |
| **6** | **PIZZA** |  | **B1** |  |  |  |
| **7** | **BURGER** |  | **B1** |  |  |  |
| **8** | **SANDWICH** |  | **B1** |  |  |  |
| **9** | **FRENCH FIRES** |  | **B1** |  |  |  |
| **10** | **OTHER FAST FOOD, *SPECIFY*:** |  |  |  |  |  |
| SECTION O: MILK PRODUCTS | | | | | | |
| **1** | **CURD** |  | **S5** |  |  |  |
| **2** | **BUTTER** |  | **TS1** |  |  |  |
| **3** | **GHEE** |  | **TS1** |  |  |  |
| **4** | **RAITHA** |  | **B2** |  |  |  |
| **5** | **OTHER 1, *SPECIFY*:** |  |  |  |  |  |

**ARE THERE ANY OTHER FOODS OR DRINKS THAT HAVE NOT BEEN MENTIONED IN PREVIOUS SECTIONS THAT YOUR EATS AT LEAST ONCE A MONTH? *SPECIFY:***

| SECTION P: OTHER FOODS | | | | | | |
| --- | --- | --- | --- | --- | --- | --- |
| **1** |  |  |  |  |  |  |
| **2** |  |  |  |  |  |  |
| **3** |  |  |  |  |  |  |
| **4** |  |  |  |  |  |  |
| **5** |  |  |  |  |  |  |
