# Supplementary material for: Life course programming of stress responses in adolescents and young adults in India: Protocol of the Stress Responses in Adolescence and Vulnerability to Adult Non-communicable disease (SRAVANA) Study
Source: Wellcome Open Res. 2018 May 10;3:56. [Version 1] doi: 10.12688/wellcomeopenres.14583.1 (PMC6039920; doi:10.12688/wellcomeopenres.14583.1)
Supplement: Supplementary file 2 [file wellcomeopenres-3-15878-s0001.tgz › 2b5fb5b7-f3e8-4b2b-94b8-b32bc1092e06.doc]

# **24- Hour Dietary Recall Questionnaire**

**Note:** Please Record **ALL** food and drink and the portion size consumed by the child in the last 24 hours.

**Ensure that you ask about drinks, fruit and any other snacks eaten during the day**

| **Time of day** | **Food Item Description** | **Food Code** | **Quantity** | **Size** |
| --- | --- | --- | --- | --- |
| **1**. After getting up |  |  |  |  |
|  |  |  |  |  |
|  |  |  |  |  |
|  |  |  |  |  |
| **2.** Breakfast |  |  |  |  |
|  |  |  |  |  |
|  |  |  |  |  |
|  |  |  |  |  |
|  |  |  |  |  |
| **3.** Between Breakfast & Lunch |  |  |  |  |
|  |  |  |  |  |
|  |  |  |  |  |
| **Time of day** | **Food Item Description** | **Code** | **Quantity** | **Size** |
| **4.** Lunch |  |  |  |  |
|  |  |  |  |  |
|  |  |  |  |  |
|  |  |  |  |  |
|  |  |  |  |  |
| **5.** Between Lunch and Dinner |  |  |  |  |
|  |  |  |  |  |
|  |  |  |  |  |
|  |  |  |  |  |
| **6.** Dinner |  |  |  |  |
|  |  |  |  |  |
|  |  |  |  |  |
|  |  |  |  |  |
|  |  |  |  |  |
| **7.** After Dinner |  |  |  |  |
|  |  |  |  |  |
