# Supplementary material for: Life course programming of stress responses in adolescents and young adults in India: Protocol of the Stress Responses in Adolescence and Vulnerability to Adult Non-communicable disease (SRAVANA) Study
Source: Wellcome Open Res. 2018 May 10;3:56. [Version 1] doi: 10.12688/wellcomeopenres.14583.1 (PMC6039920; doi:10.12688/wellcomeopenres.14583.1)
Supplement: Peer review history

# Peer review: Referee response for version 1

**DOI:** 10.21956/wellcomeopenres.15878.r33412

**Published:** 2018-07-06

**Authors:** Giridhara R. Babu

**Affiliations:** 1 Indian Institute of Public Health (IIPH) Bangalore, Public Health Foundation of India, Bangalore, Karnataka, India

The protocol of the “Stress Responses in Adolescence and Vulnerability to Adult Non-communicable disease (SRAVANA) study” is written well.  

 The objectives are outlined well. The authors propose using the validated Trier Social Stressor scale apart from several psychological and biological measures. 

 My comments are noted below:-

 HPA-axis activation and inhibition involves several psychosocial stressors and buffers ^1^^-^^2^.While sufficient details are provided on how the investigators measure stressors including cortisol levels, it would be useful to know how buffer mechanisms are measured ^3^^-^^4^. For example, evidence suggests that social support ^5^^-^^6^, secure attachment ^5^ and social identity ^7^ buffers stress response including and cortisol levels ^8^.

 The investigators mention that they propose to develop a socio-cognitive intervention method for stress management. It is useful to know whether the data for such an intervention is generated from the proposed study. If so, the assessments for cognitive function are explained as part of the secondary outcomes. However,  details on how social environment is measured are missing. The authors may clarify regarding this.

 Given that this study is multi-centric and involves three Indian birth cohorts; it will be useful to know how the team is planning to integrate the efforts of the different cohorts. A table mentioning common components in all the three cohorts, and specific examinations within individual cohorts can be useful. Also, it will be useful to know how the variations in population composition, outcomes and initial assessments amongst the three cohorts are accounted for.

 Minor:-

 Understanding the role of prenatal nutritional intervention in reducing physiological stress responses in the adolescent offspring is a worthy objective. The readers will get benefited if the theory and evidence regarding the mechanisms involved between nutrition and physiological stress are explained. This will also justify the role of testing nutritional supplementation during adolescence/young adulthood is associated with improved stress responses in young adults exposed to maternal nutritional deficiency.

 The authors mention that all assays will be carried out using standard protocols. It will be useful to provide brief details about the protocols they will follow. Alternatively, they can upload the links to manuals or recommendations they are following.  For example, it is useful to know how many samples of salivary cortisol will be collected? At what time of the day are these collected?

I have read this submission. I believe that I have an appropriate level of expertise to confirm that it is of an acceptable scientific standard.

## References

[1] SelyeH: Homeostasis and heterostasis. *Perspect Biol Med*.1973;16(3) :441-5 470507310.1353/pbm.1973.0056

[2] JacksonJSKnightKMRaffertyJA: Race and unhealthy behaviors: chronic stress, the HPA axis, and physical and mental health disparities over the life course. *Am J Public Health*.2010;100(5) : 10.2105/AJPH.2008.143446 933-9 10.2105/AJPH.2008.143446 19846689PMC2853611

[3] SmeetsTDziobekIWolfOT: Social cognition under stress: differential effects of stress-induced cortisol elevations in healthy young men and women. *Horm Behav*.2009;55(4) : 10.1016/j.yhbeh.2009.01.011 507-13 10.1016/j.yhbeh.2009.01.011 19470361

[4] von DawansBFischbacherUKirschbaumCFehrEHeinrichsM: The social dimension of stress reactivity: acute stress increases prosocial behavior in humans. *Psychol Sci*.2012;23(6) : 10.1177/0956797611431576 651-60 10.1177/0956797611431576 22593119

[5] DitzenBSchmidtSStraussBNaterUMEhlertUHeinrichsM: Adult attachment and social support interact to reduce psychological but not cortisol responses to stress. *J Psychosom Res*.2008;64(5) : 10.1016/j.jpsychores.2007.11.011 479-86 10.1016/j.jpsychores.2007.11.011 18440400

[6] HostinarCEJohnsonAEGunnarMR: Parent support is less effective in buffering cortisol stress reactivity for adolescents compared to children. *Dev Sci*.2015;18(2) : 10.1111/desc.12195 281-97 10.1111/desc.12195 24942038PMC4270957

[7] HäusserJKattenstrothMvan DickRMojzischA: “We” are not stressed: Social identity in groups buffers neuroendocrine stress reactions. *Journal of Experimental Social Psychology*.2012;48(4) : 10.1016/j.jesp.2012.02.020 973-977 10.1016/j.jesp.2012.02.020

[8] KikusuiTWinslowJTMoriY: Social buffering: relief from stress and anxiety. *Philos Trans R Soc Lond B Biol Sci*.2006;361(1476) : 10.1098/rstb.2006.1941 2215-28 10.1098/rstb.2006.1941 17118934PMC1764848

# Peer review: Referee response for version 1

**DOI:** 10.21956/wellcomeopenres.15878.r33254

**Published:** 2018-06-19

**Authors:** Candace M. Raio

**Affiliations:** 1 Center for Neural Science, New York University, New York, NY, USA

This is a proposal for a study that aims to measure acute stress reactivity in a large sample of adolescents drawn from three birth cohorts in India (Mysore Parthenon cohort; SARAS KIDS prenatal intervention cohort; and PMNS cohort). The authors nicely motivate the study based on the extant literature pointing to maternal nutrition as an important factor that shapes risk for NCDs later in life. One pathway, they convincingly argue, through which this might occur is by programming of stress responses. Therefore, they propose to examine how early life factors confer risk for dysregulation of stress responses that may later give rise to NCDs.

 Their key objectives are outlined clearly, and include identifying prenatal factors (e.g. maternal nutrition/metabolic health) that predict stress responses in adolescents, how these response relate to psychological and cardiovascular health in adolescents, how nutritional interventions (both to pregnant mothers and their offspring) relate to improvements in adolescent stress reactivity and, finally, how socio-cognitive intervention models can help reduce stress responses in these adolescents.

 The authors propose using the Trier Social Stressor, which is well-validated and reliable, and propose collecting both neuroendocrine (cortisol) and autonomic arousal (cardiovascular) measures to index stress reactivity. A number of other psychological and biological measures will be collected, including subjective stress, cognitive function, blood pressure, etc. Imaging and genetics will be collected on a subset of participants.  

 The sample sizes are large, well-powered and sample from different age ranges. The authors propose that ~250 20-year old participants from the Parthenon cohort, 300 adolescents (half from mothers who underwent a nutritional intervention and half who were controls) from the MMNP, and a subset of young adults (~150) involved in an ongoing nutritional supplementation program, will undergo the acute stressor. The authors clearly describe their psychological, cardiovascular, metabolic and other behavioral measures.

 The data will be useful to acquire a better understanding of how prenatal nutrition shapes trajectories of stress response in offspring and will facilitate our understanding how these responses may confer vulnerability to NCDs. 

 I would suggest the following:

 (1) That the authors should indicate the age and gender of the evaluators for the TSST, as this can influence stress reactivity in children and adolescents (i.e., are these evaluators 'peers', which we know are highly salient to adolescents, or are they adult observers?). 

 (2) Given that uncontrollability and unpredictability are seminal features of the stress response, the authors may want to consider adding a measure of both. For example, the Intolerance of Uncertainty Scale or the updated Locus of Control Inventory (4th ed.) could provide important information regarding what feature of the Trier stressor is driving their individual stress reactivity. These are brief and easy to administer.

 (3) Will the database be made publicly available after the study is complete? Currently, the authors mention their data will be disseminated through publications but it would be good to know if this important data set will be available to other researchers/collaborators. 

 (4) I assume both genders will be represented in the data set. The authors should consider documenting female menstrual cycle in the samples of young adults and any other hormonal medication that can affect cortisol responses.

I have read this submission. I believe that I have an appropriate level of expertise to confirm that it is of an acceptable scientific standard.
